# Tailoring Photoprotection of Polylactide with New Isobornyl Derivatives of Phenol and Aniline

**DOI:** 10.3390/polym15092141

**Published:** 2023-04-29

**Authors:** Vladimir A. Belyi, Ivan M. Kuzivanov, Irina V. Fedorova, Olga A. Shumova, Evgeniy M. Tropnikov, Elena I. Istomina, Irina Yu. Chukicheva, Aleksandr V. Kuchin

**Affiliations:** 1 Institute of Chemistry of FRC Komi SC UB RAS, 167000 Syktyvkar, Russia; 2 Institute of Geology of FRC Komi SC UB RAS, 167000 Syktyvkar, Russia

**Keywords:** photodegradation, environmentally friendly polymers, polylactide

## Abstract

This article is devoted to the development of new photostabilizers for polylactide (PLA), a polymer that is an environmentally friendly alternative to polymers and is based on fossil raw materials. We have elucidated the role of the reaction center of two potential PLA photoprotectors: *N*-isobornylaniline and 2-isobornylphenol, in reactions occurring in a polymer matrix under the action of UV-C radiation. PLA samples with the photostabilizers were irradiated under a wavelength of 253.7 nm for 4, 8 and 12 h. The effectiveness of the photostabilizers was evaluated based on FTIR spectrometric data, ^1^H and ^13^C NMR, scanning electron microscopy and simultaneous thermal analysis (TG-DSC). Both stabilizers led to the protection of ester bonds between monomer units of PLA. However, 2-isobornylphenol proved to be more effective at a concentration of 0.05 wt.%, while the optimal concentration of *N*-isobornylaniline was 0.5 wt.% by weight. TG-DSC showed that the addition of *N*-isobornylaniline led to an increase in PLA resistance to thermal decomposition; the temperature of the onset of weight loss increased by 2.8 °C at 0.05 wt.% and by 8.1 °C at 0.5 wt.% of *N*-isobornylaniline. The photoprotector 2-isobornylphenol, on the contrary, reduced the thermal stability of PLA.

## 1. Introduction

The problem of polymer waste has become global in the last two decades [1]. Scientific research in this area is focused on the ways to recycle polymers, the rejection of fossil raw materials and the transition to renewable alternatives, as well as on microplastics’ potential harm to the life and health of people and marine fauna [2,3].

Polymers quite often undergo photo-oxidative and thermal-oxidative degradation both during use and during molding and recycling. To slow down the process of oxidative degradation, small amounts of antioxidants (AOAs) are added to the polymer composition, usually at 0.05–0.5 wt.%. Hindered phenols, amines, or organophosphorus compounds can be used as AOAs. However, given the variety of applications of polymers and the increasing requirements for the safety and quality of materials, there is a need to develop and study new AOAs and to study their behavior in new polymer matrices as well as under specific operating conditions. The challenges of using AOAs that need to be addressed include increasing the temperature stability of AOAs, increasing the compatibility of the polymer and stabilizer, and reducing toxicity, which is especially important when they are used for food packaging and medical applications. Therefore, scientific interest in the development of new thermal and photostabilizers for polymers that can extend the service life of products and preserve the performance characteristics of polymers during recycling is not decreasing [4,5].

Among synthetic polymers, polylactide (PLA), an aliphatic polyester of lactic acid, has the greatest potential for expanding the scale and scope of application due to its biocompatibility and extensive plant resource base [6,7,8]. However, certain properties of PLA require improvement, in particular its low thermal-oxidative stability and resistance to photodegradation [9,10]. A very popular trend in recent years in PLA research is associated with the development of controlled photodegradation [11,12,13,14,15]. Thus, researchers and consumers are interested in a whole set of tools for the targeted regulation of the photosensitive properties of polylactide.

Shortwave radiation in the 100–280 nm wavelength range (UV-C) is the most damaging type of UV radiation for polymer products. Ozone in the upper atmosphere absorbs solar UV radiation, but as the ozone layer becomes thinner due to environmental concerns, the protective filtering power of the atmosphere gradually decreases. Already, studies show that the intensity of UV-C at 245 nm penetrating to the Earth’s surface reaches 2.54 mW/cm^2^ in some regions, which can cause erythema on light human skin after just a few seconds of exposure. As for polymers, this intensity of UV-C leads to a decrease in the molecular weight of PLA by more than 4 times after 24 h of irradiation [16,17]. Accordingly, scientific interest is growing in the development of new thermal and photostabilizers for polymers that can extend the service life of products and preserve the performance characteristics of polymers during recycling [4,5]. It is possible to improve the properties of PLA with the help of modifying additives.

Unfortunately, most of the technically significant polymer-modifying additives are currently produced from fossil resources. Extractable components of plant biomass such as terpenes can be an excellent sustainable feedstock for the synthesis of polymer-modifying additives. Biomass-derived compounds are now attracting growing interest from researchers for the synthesis of various derivatives with a broad range of applications [18,19].

The newly tested classes of modifying additives for polymers are based on the combination of a terpene fragment and a phenolic or aniline fragment with the same chemical structure as a free radical scavenger [20,21,22,23]. Due to the ability to effectively inhibit the radical chain oxidation processes of hydrocarbon substrates and due to their low toxicity, alkylated phenols are widely used as stabilizers in the petrochemical, polymer, and food industries [24]. Therefore, it is of theoretical and applied interest to compare the reactivity of new *ortho*-alkylated phenols with an isobornyl substituent and *N*-isobornyl aniline derivatives. The development of new compounds based on by-products of wood processing, including terpenes, requires the identification of structure–property patterns for new applications.

**Figure 1:**
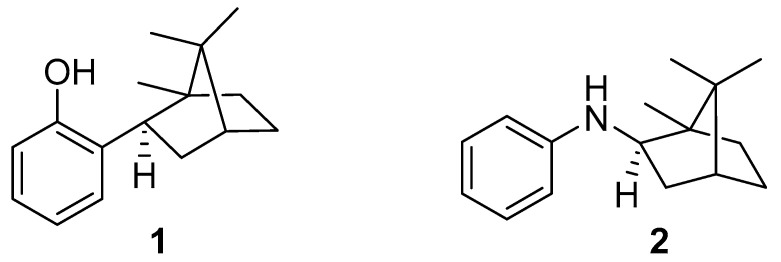
Polylactide photostabilizers: (**1**)—2-isobornylphenol (IBP); (**2**)—*N*-isobornylaniline (NIA).

The purpose of this work is to elucidate the role of the reaction center of the new potential polylactide photoprotectors 2-isobornylphenol (IBP) and *N*-isobornylaniline (NIA) (Figure 1) in reactions occurring in a polymer matrix under the action of UV-C radiation with a maximum intensity at 253.7 nm. These compounds are very close in molecular weight and have the same terpene moiety, but they differ in their theoretical mechanism of action in protecting the polymer. The comparative study makes it possible to reveal the role of the reaction center in the protection of the polymer matrix from ultraviolet radiation. The effect of the structural features of the new stabilizers on their activity was studied using FTIR spectrometric data, ^1^H and ^13^C NMR, scanning electron microscopy and TG-DSC. To our knowledge, no previous research has tested terpene-based antioxidants as polymer photoprotectors. The structure–property relationships of terpene-based additives in polymers will help to optimize their structural design during further syntheses and improve the effectiveness of the additives.

## 2. Materials and Methods

In this study, PLA Ingeo™ Biopolymer 4043D manufactured by Naturalworks (USA) was used. Substances studied as photostabilizers (1 and 2, see Figure 1), were synthesized by the methods described in [25,26]. The preparation of PLA films with a certain concentration of a photostabilizer was carried out as follows: 1.3 g of PLA with a calculated weight of a photostabilizer was dissolved in 15 mL of dichloromethane; 1 mL of the resulting solution was applied to a glass plate 25.4 × 76.2 mm in size. Next, the sample was dried at 60 °C for 2 h. The most common concentration of photostabilizers in polymer studies is 0.5 wt.%, judging by the literature. However, in order to identify primary evidence of the influence of the photostabilizer concentration, in this study we used two weight concentrations of the photostabilizers in the PLA: 0.05 wt.% and 0.5 wt.%. These concentrations allowed us to compare the results with the literature data and to reveal the effect of concentration on photoprotection activity.

The experimental setup for irradiation was a metal chamber with a radiation source inside—a 25 W UV-C quartz lamp with a wavelength maximum intensity at 253.7 nm; the temperature in the chamber was 29 °C. The distance between the irradiated sample and the radiation source was 40 mm. Plates with PLA samples were placed on the experimental irradiation setup for 4, 8 or 12 h.

Fourier transform infrared spectroscopy (FTIR) of the original and irradiated PLA samples was performed with an IR Fourier spectrometer IR Prestige-21 (Shimadzu) equipped with a DLATGS detector to analyze changes in functional groups and bonds caused by UV-C irradiation. The transmission spectra were obtained in the diffuse reflection mode. Spectra were recorded at a range of 4000–700 cm^−1^ at the resolution of 4 cm^−1^, and the number of scans was set to 20. The data were processed using Shimadzu software. The spectra were integrated quantitatively in Origin 6.1.

The ^1^H and ^13^C JMOD NMR spectra of the PLA samples before and after irradiation were recorded in 5 mm tubes on a Bruker Avance II 300 spectrometer. Around 0.1 g of a polymer sample was dissolved in 0.6 cm3 of deuterated chloroform (CDCl_3_, >99.9%, Solvex). The spectra were referenced to the residual signals of chloroform (7.26 ppm for ^1^H and 77.5 ppm for ^13^C spectra). The spectra were processed using the Spinsolve 1.19.2 program. The obtained spectra were interpreted according to literature data [27,28].

To study the morphology of the surface and cracks of the irradiated polymer samples, scanning electron microscopy was used with a Vega3 SBU (TESCAN) microscope. The following conditions were used: elastic electron scattering mode, accelerating voltage of 10 kV.

The study of the thermal properties of the polymer samples was carried out by the method of simultaneous thermal analysis (TG-DSC) with a METTLER TOLEDO TGA/DSC 3+ thermal analyzer. The measurements were carried out in a dynamic mode at a temperature range of 25–600 °C and at a heating rate of 5 °C/min in platinum crucibles in air. The measurement error was ±1%.

## 3. Results and Discussion

**Figure 2:**
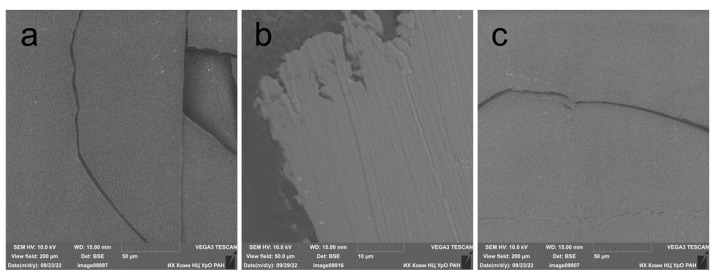
Microphotographs of surface of PLA films after 4 h of UV irradiation: (**a**) without AOA additive; (**b**) with the addition of 0.05% wt. IBP; (**c**) with the addition of 0.05% wt. NIA.

Polylactide is a polymer that is extremely sensitive to UV radiation, which significantly reduces the mechanical properties of PLA packaging and medical materials [29]. The impact of radiation on the UV-C range of the spectrum for the studied PLA films without additives and with additives of stabilizers for four hours led to a significant increase in the fragility of the material. Figure 2 shows the SEM micrographs of the irradiated films. The film before irradiation had elasticity, strength and a smooth surface. However, after irradiation, it crumbles into fragments with only a slight mechanical impact (Figure 2a). The cracks are linear, with smooth edges, which indicates a loss of elasticity of the polymer. A similar pattern of destruction was observed on the PLA film with the addition of 0.05% wt. NIA (Figure 2c) after 4 h of irradiation. The film with the addition of IBP retained its elasticity, and the edges of the studied film fragment were uneven, which reveals a completely different nature of damage under mechanical action. These SEM micrographs show the significant difference in the photoprotective activity of IBP and NIA.

**Figure 3:**
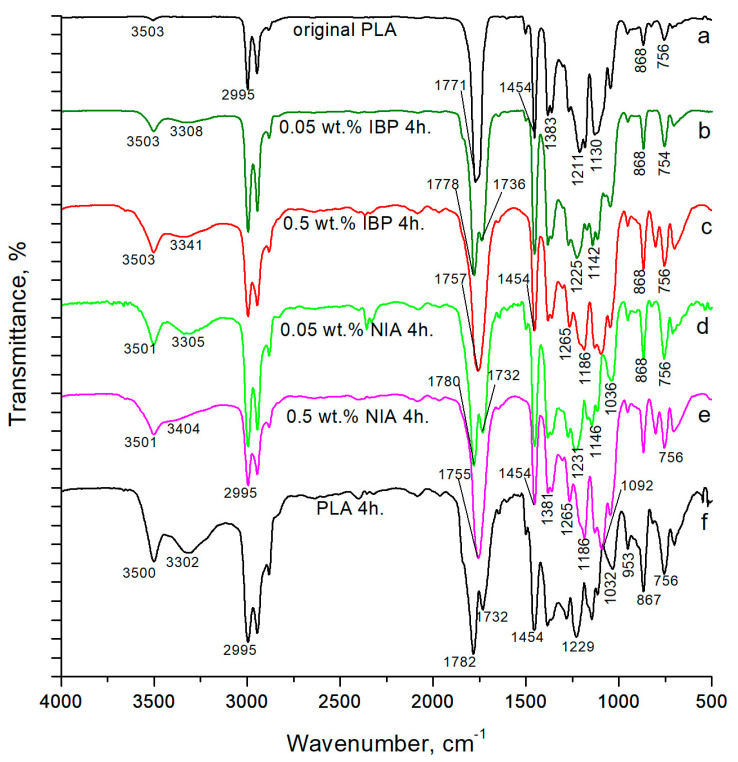
FTIR spectra of PLA samples: (**a**) original PLA without AOA; (**b**) PLA with 0.05% wt. IBP after 4 h of irradiation; (**c**) PLA with 0.5% wt. IBP after 4 h of irradiation; (**d**) PLA with 0.05% wt. NIA after 4 h of irradiation; (**e**) PLA with 0.5% wt. NIA after 4 h of irradiation; (**f**) PLA without AOA after 4 h of irradiation.

Changes in the chemical structure of the polymer under radiation became apparent when comparing the FTIR spectra of the PLA samples (Figure 3). Table 1 shows the assignment of absorption bands in the FTIR spectra of the PLA samples [30,31]. The terminal hydroxyl groups of the initial polymer before irradiation showed a relatively small absorption peak at 3500 cm^−1^. However, with an increase in the time of photooxidative degradation, an increasing number of ester bonds in the polymer chain were broken and, as a result, the number of terminal hydroxyl and carboxyl groups increased. At the same time, the absorption in the region of the FTIR spectrum of 3600–3100 cm^−1^ increased significantly. The absorption range in the FTIR spectra of 1200–1000 cm^−1^, on the contrary, demonstrated a decrease in the absorption intensity.

The UV degradation effect on the PLA led to the formation of new absorption bands in the FTIR spectra; the band at 1732 cm^−1^ corresponded to the –C=O stretching vibrations of the carboxyl groups, and the weak band with a maximum at 1843 cm^−1^ attributed to the anhydride groups appeared after 4 h of UV-C treatment [32,33,34].

The terminal hydroxyl groups of the initial polymer before irradiation showed a relatively small absorption peak at 3500 cm^−1^. All spectra of the irradiated samples showed an increase in this absorption peak. At the same time, the photoprotection from 0.05 wt.% of IBP caused a noticeably lower increase in absorption in the 3600–3100 cm^−1^ region of the FTIR spectrum, compared to with 0.5 wt.% of IBP (Table 2).

The introduction of 0.05% wt. of the photoprotecting additives to the polymer structure primarily resulted in the protection of the ester bonds between the monomeric units of the PLA. The absorption range of the ester bonds in the FTIR spectra of the PLA (Figure 3) (1200–1000 cm^−1^) showed more than a 50% decrease in the relative absorption intensity after four hours of irradiation—from 0.274 to 0.118. However, in the sample with the addition of 0.05% wt. of IBP, the relative absorption intensity in this region decreased by only 15%—from 0.274 to 0.232. When using NIA in the same concentration, the relative absorption intensity in this region decreased by 46%—from 0.274 to 0.148. However, it should be noted that 0.5 wt.% of NIA demonstrated the same level of protection for PLA ester bonds as 0.05 wt.% of IBP; the relative absorption intensity in the region of 1200–1000 cm^−1^ decreased by 15%, from 0.274 to 0.235 (Table 2).

From an applied point of view, IBP belongs to the group of polymer UV stabilizers in the class of sterically hindered phenols, and NIA is a hindered amine light stabilizer. It is known that at the first stage of stabilization, the hydrogen atom of the phenolic group of sterically hindered phenols passes to the radical species that arise in the polymer. At the second stage, the hydrogen atom is donated in the α-position, which leads to the formation of a quinoid structure [20]. The photostabilizing ability of the hindered amine light stabilizer is based on the formation of nitroxyl radicals under radiation, which are able to recombine with the polymer radical and thereby interrupt the degradation of the polymer chain [4]. However, as can be seen from the obtained FTIR spectroscopy data on the preservation of ester bonds in PLA macromolecules, the photoprotective activity of both NIA and IBP is highly dependent on their concentration in the polymer. NIA is very much inferior to IBP in terms of photoprotective ability at a concentration of 0.05 wt.%; however, it performed well at a concentration of 0.5 wt.%.

**Figure 4:**
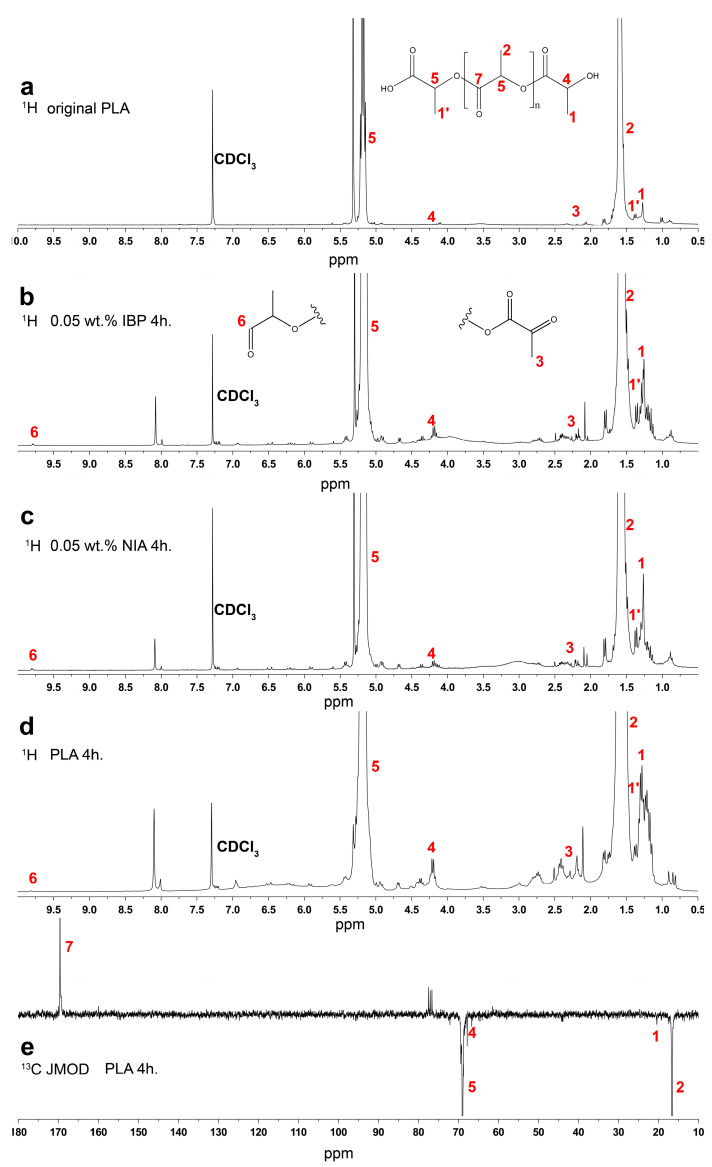
^1^H spectra of original (**a**) and irradiated (**b**–**d**) PLA; ^13^C JMOD NMR spectrum of irradiated PLA (**e**).

Figure 4 shows the ^1^H spectrum of the original PLA (Figure 4a) and the ^1^H and ^13^C JMOD NMR spectra of the samples irradiated for 4 h (Figure 4b–f). It can be noted that in addition to the major signals of the polymer structure, the spectrum of the irradiated PLA samples (Figure 4b–d) clearly shows the increased signals of the terminal methyl groups of the PLA in the areas of the chemical shifts at 1.0–1.4 ppm and 2.0–2.5 ppm. The signals in the region of 1.43–1.49 ppm are originated from CH_3_ groups belonging to carboxyl terminal moieties [35]. The peaks in the region of 4.0–4.5 ppm are presumably associated with the CH of the terminal lactoyl groups. The appearance of these terminal groups was also supported by the signal at 20 ppm in the ^13^C JMOD NMR spectrum of the irradiated sample (Figure 4e) [36] and the signal at 66.7 ppm (CH groups of hydroxyl terminal moiety of PLA) [35]. Signals indicating the appearance of new terminal aldehyde groups were found at 9.8 ppm in the ^1^H spectra. The increase in intensity of the signals of these terminal groups in the ^1^H NMR spectra is caused by the breaking of ester bonds in the polymer chain and points to a decrease in the molecular weight of the polymer after irradiation.

**Figure 5:**
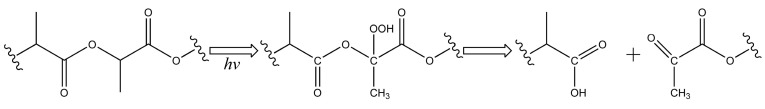
Photo-oxidative degradation of PLA according to the formation of a hydroperoxide derivative followed by the formation of carboxyl groups, as well as a diketone.

It is known that UV radiation in the range of 220–280 nm is absorbed by the PLA carbonyl groups and causes the n-π* electron transition. This excited state of the electron is capable of inducing a macromolecular chain scission reaction according to the Norrish type II mechanism [6]. The result of this reaction should be an appearance of new hydroxyl and vinyl groups. However, there were no significant signals from vinyl groups in the region of 6.0–6.7 ppm in the ^1^H NMR spectra of the irradiated samples. In this case, the reaction presented in Figure 5 is assumed to be more probable. In accordance with this reaction, the formation of a hydroperoxide derivative is possible, followed by the formation of carboxyl groups, as well as a diketone [37].

Another aspect that revealed the influence of structure on the reactivity of the studied isobornyl derivatives in the PLA matrix is their thermal behavior in the air atmosphere.

**Figure 6:**
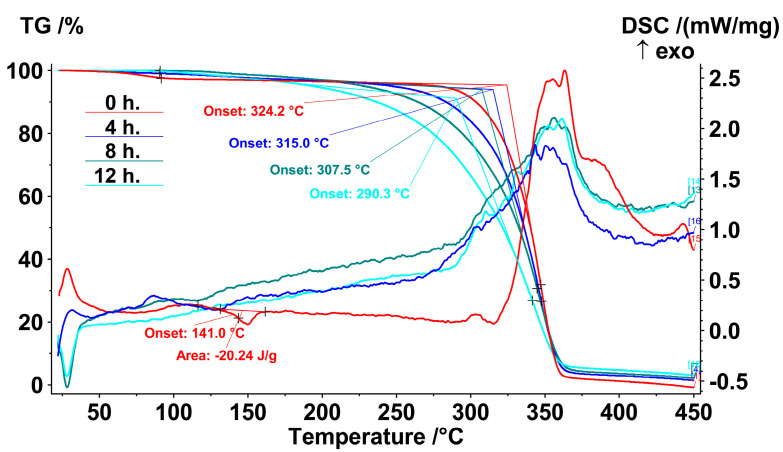
TG-DSC thermograms of PLA samples without AOA after 0, 4, 8 and 12 h of UV-C irradiation.

The characterization of thermoplastic materials and improving thermal stability are keys for recycling polymers and for additive manufacturing development. Important thermal material properties can be revealed by thermal analysis [38,39]. TG-DSC was used to study the thermal degradation of PLA without additives irradiated under UV-C radiation (Figure 6), as well as for PLA samples with the addition of AOAs (IBP or NIA).

It should be noted that the melting peak at 141.0 °C was observed only for the initial PLA. Even just 4 h of UV-C exposure led to the disappearance of this peak, which meant greater changes in PLA structure than that revealed in [40] with the same UV-C wavelength. After 275 °C, an exothermic effect began to develop, which accompanied the intense weight loss. The temperatures at the onset of thermal decomposition were calculated by the extrapolation of linear segments in the TGA curves, according to the standard ISO 11358-1. The duration of irradiation led to a decrease in the temperature at the onset of polymer decomposition from 324.2 °C to 290.3 °C after 12 h of irradiation.

**Figure 7:**
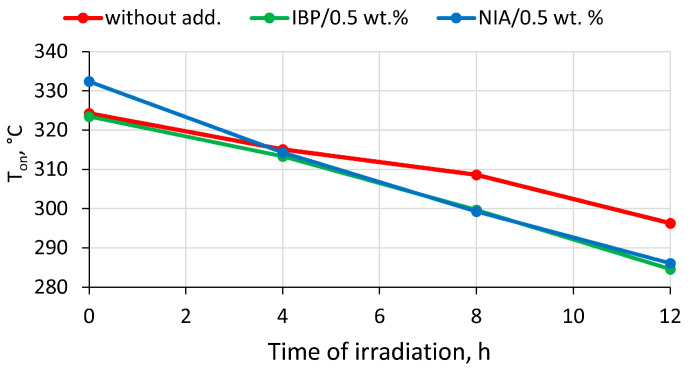
Dependence of the temperature of the onset of thermal degradation (T_on_) on the time of UV-C irradiation of PLA samples: initial PLA without AOA; PLA with 0.5 wt.% IBP; PLA with 0.5 wt.% NIA.

The addition of 0.5 wt.% of NIA increased the onset temperature of polylactide decomposition by approximately 8 °C. The insertion of 0.5 wt.% of the IBP additive had almost no effect on the temperature at the start of decomposition (Figure 7). However, neither IBP nor NIA had a thermoprotective effect on the irradiated samples. The assumption for this may be that their protective action was exhausted by irradiation. Moreover, the irradiated structure of polylactide in the presence of the additives IBP and NIA began to decompose at a lower temperature; for example, samples irradiated for 12 h began to decompose at a temperature of almost 12 °C lower than that of a similar irradiated sample without additives.

From these results, it becomes obvious that, along with the photoprotective effect on the polylactide, IBP does not have thermoprotective properties and causes a decline in the temperature at the onset of thermal degradation. However, the question remains regarding the correlation between the concentrations of IBP and NIA and their reactivity in the polymer matrix upon heating, since it was shown above that the photoprotective properties of these low-molecular-weight additives strongly depend on the applied concentration in the polymer.

**Figure 8:**
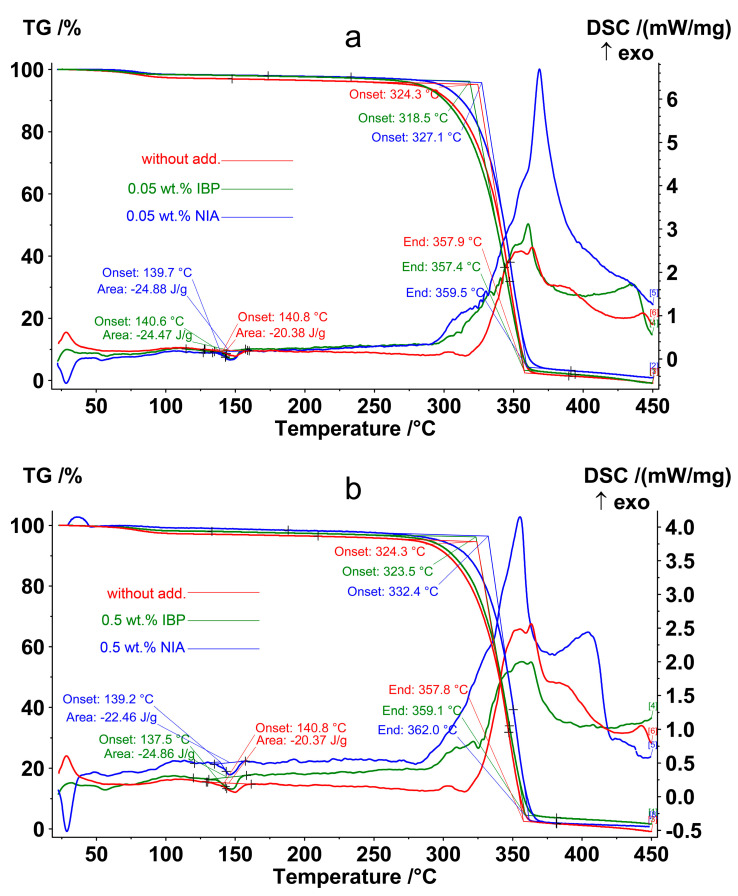
TG-DSC thermograms of non-irradiated PLA samples: (**red**)—PLA without AOA; (**green**)—PLA with 0.05 wt.% (**a**) and 0.5 wt.% (**b**) IBP; (**blue**)—PLA with 0.05 wt.% (**a**) and 0.5 wt.% (**b**) NIA.

The IBP concentration of 0.05 wt.% was the most effective for photoprotection, since it showed a high level of preservation of ester bonds between monomeric units (Table 2). The effect of an AOA concentration of 0.05 wt.% on the thermal decomposition of non-irradiated PLA is shown in Figure 8a and Table 3, where it is compared with the IBP and NIA concentration of 0.5 wt.% (Figure 8b).

All non-irradiated samples showed a melting peak at almost the same temperature and enthalpy with a slight deviation, 139.6 ± 1.3 °C and −23.4 ± 2.0 J/g, respectively (Table 3). At a temperature of 324.3 °C, the intense thermal-oxidative destruction of the initial PLA began, accompanied by weight loss and an exothermic effect. The effect of adding 0.05% IBP was quite unexpected—the temperature at the onset of the thermo-oxidative degradation of the PLA with IBP decreased to 318.5 °C, despite the fact that this concentration was optimal for the photoprotective properties of IBP. The use of NIA as an additive, on the contrary, led to an increase in the thermal-oxidative stability of the polymer, with the temperature at the onset of weight loss equal to 327.3 °C and 332.4, at 0.05 wt.% and 0.5 wt.%, respectively (Table 3). Thus, a concentration of 0.05 wt.% NIA increased the temperature at the onset of PLA decomposition by 2.8 °C, and a concentration of 0.5 wt.% NIA increased the temperature at the onset of PLA decomposition by 8.1 °C.

One of the assumptions that explains the high photoprotective activity of IBP, along with its weak thermos-oxidative protection, may be the predominance of the UV absorption mechanism during UV protection over the mechanism of radical neutralization described above. The UV absorption mechanism is implemented by converting the energy of absorbed photons by means of proton transfer between the groups involved in the intra- and intermolecular hydrogen bonds: =O…HO– or =O…HN<. The reverse reaction is exothermic, and heat is dissipated within the polymer matrix. The question of the influence of this mechanism on the interaction of NIA or IBP with the PLA polymer matrix requires an additional study.

## 4. Conclusions

Polylactide is one of the most environmentally promising polymeric materials that could replace fossil-based polymers. We have studied the influence of the nature of the reaction center during the photoprotective action of new classes of compounds, namely, terpenophenols and terpene-substituted anilines containing an isobornyl fragment. IBP and NIA are obtained by the alkylation of phenol and aniline with camphene, a naturally occurring monoterpene. Improving the resistance of PLA to ultraviolet radiation was achieved in this work by introducing an additive of IBP into the polymer structure at a concentration of 0.05% by weight.

The increasing brittleness of a polymer material under the action of ultraviolet radiation is the most important problem from an applied point of view. It was found that the addition of IBP at a concentrations of 0.05% by weight prevents the cracking of the polymer film after 4 h of UV-C exposure. The analysis of the FTIR spectra of irradiated PLA with the addition of IBP showed that the studied terpene phenol resists depolymerization and retains the ester bonds of the polymer. It was shown by TG-DSC that the addition of NIA led to an increase in the resistance of PLA to thermal decomposition. IBP, on the contrary, reduced the thermal stability of PLA.

The manifestation of the good photoprotective activity of IBP, in combination with the weakening of the thermal-oxidative protection of the polymer, can be explained by the predominance of the UV absorption mechanism in the protection of the polymer from ultraviolet radiation over the mechanism of the neutralization of free radicals. Thus, the use of IBP as a photoprotective additive will extend the service life of polylactide plastic products that are exposed to aggressive UV-C radiation.

The manifestation of the good photoprotective activity of IBP, in combination with the weak thermal-oxidative protection of the polymer, can be explained by the predominance of the UV absorption mechanism in the protection of the polymer from ultraviolet radiation over the mechanism of the neutralization of free radicals. Thus, the use of IBP as a photoprotective additive will extend the service life of polylactide plastics that are exposed to aggressive UV-C radiation.

## Figures and Tables

**Table 1:** Assignment of absorption bands in FTIR spectra of PLA.

Wave Number, cm^−1^	Band Assignment
3600–3100	stretch vibrations–OH
2995.4; 2945.3	stretch vibrations –CH–
1774.5	stretch vibrations –C=O
1454.3	deformation –CH_3_
1382.9; 1361.7	deformation –CH–
1217.1	deformation –C=O
1183.4; 1136.1; 1114.9	stretch vibrations –C–O–
1047.4	deformation –OH
952.8; 868.0	stretch vibrations –C–C–

**Table 2:** Relative integrated intensities I_x_/I_4000-400_ of absorption intervals (x) in FTIR spectra of PLA.

Wave Numbers of Intervals (x), cm^−1^	Initial PLA	IBP 0.05 wt.% 4 h.	IBP 0.5 wt.% 4 h.	NIA 0.05 wt.% 4 h.	NIA 0.5 wt.% 4 h.	PLA 4 h.
	I_x_/I_4000–400_					
3600–3100	0.008	0.057	0.122	0.113	0.111	0.164
1200–1000	0.274	0.232	0.207	0.148	0.235	0.118

**Table 3:** Parameters of thermal-oxidative destruction of PLA samples by TG-DSC.

PLA Additive/C, wt.%	T_melt_, °C	∆H_melt_, J/g	T_on_, °C	T_off_, °C
IBP/0.05	140.6	−24.47	318.5	357.4
IBP/0.5	137.5	−24.86	323.5	359.1
NIA/0.05	139.7	−24.88	327.1	359.5
NIA/0.5	139.2	−22.46	332.4	362.0
without add.	140.8	−20.38	324.3	357.9

## Data Availability

The raw data required to reproduce these findings can be shared. Readers are encouraged to communicate with the corresponding author for more information.
